# Internalized Weight Stigma and Weight Discrimination: Associations with Quality of Life and Psychosocial Impairment in a Sample Living with Food Insecurity

**DOI:** 10.3390/ijerph20247147

**Published:** 2023-12-06

**Authors:** Sabrina E. Cuauro, Natalia Santos, Estefania Andrade, Anoushka W. Dani, Saivone N. Sanchious, Savannah C. Hooper, Carolyn Black Becker

**Affiliations:** 1 Department of Psychology, Trinity University, San Antonio, TX 78212, USA; nsantos@trinity.edu (N.S.); andradestefi@gmail.com (E.A.); anoushka.dani@assumption.edu (A.W.D.); ssanchious2000@gmail.com (S.N.S.); cbecker@trinity.edu (C.B.B.); 2 Department of Psychological Sciences, Rice University, Houston, TX 77005, USA; 3 Department of Psychiatry and Human Behavior, Brown University, Providence, RI 02912, USA; 4 Department of Psychological and Brain Science, University of Louisville, Louisville, KY 40292, USA; savannah.hooper@louisville.edu

**Keywords:** weight discrimination, food insecurity, quality of life, psychosocial impairment

## Abstract

Research suggests that experiencing weight discrimination is associated with a lower quality of life and poor psychological and physical health. However, much of the existing weight discrimination literature has neglected under-represented groups. Little is known about how the experience of weight discrimination affects quality of life and eating/weight-related psychosocial impairment in those living with food insecurity. The present study investigated the associations of weight discrimination and eating/weight-related psychosocial impairment and quality of life. We examined internalized weight stigma and several psychological indicators as potential mediators. Participants (*N* = 1085) who were recruited from a local food bank completed a questionnaire assessing food insecurity, weight discrimination, internalized weight stigma, eating disorder pathology, anxiety, depression, eating/weight-related psychosocial impairment, and quality of life. Overall, almost one in four participants reported experiencing weight discrimination. Our serial mediation models indicated that increased experiences of weight discrimination were associated with greater internalized weight stigma and psychopathology, which were in turn associated with lower quality of life and greater eating/weight-related psychosocial impairment. Thus, experiencing weight discrimination may negatively impact quality of life and eating/weight-related psychosocial impairment through its effect on mental health. It is imperative to address the negative effects of the widespread discrimination of people based on their weight.

## 1. Introduction

Weight stigma, also known as weight bias, encompasses prejudice, stereotypes, and discrimination towards people living in higher-weight bodies [1,2,3]. Weight discrimination refers to the actions and behaviors stemming from weight-related prejudices and stereotypes [1]. Weight discrimination is prevalent in workspaces, healthcare, educational institutions, transportation, and amongst family and friends [4]. This type of discrimination affects individuals across race, age, and gender, and its incidence has steadily increased by almost 70% over the course of 10 years [5]. The prevalence of lifetime experiences of weight discrimination is 10.3% for women and 4.9% for men [4]. For individuals of a higher weight (BMI of ≥35), the prevalence of weight discrimination is 40% [4]. Examples of weight discrimination include receiving inferior healthcare, being called derogatory names, or experiencing harassment [6]. Despite its widespread occurrence, harmful effects, and rapid growth, weight discrimination receives little to no attention from bias-prevention programs and federal laws compared to other forms of social stigma [4,7].

Increased and repeated experiences of weight discrimination can lead to detrimental effects on physiological and psychological functioning. Importantly, experiencing all forms of weight stigma, including discrimination, can initiate a positive feedback loop whereby increased levels of stress-induced cortisol secretion in the body lead to increased eating behavior, resulting in weight gain [3]. Increased weight then leads to greater probability of experiencing weight discrimination, perpetuating this vicious cycle [3]. Weight discrimination does not only increase risk of weight gain: individuals of higher weight who experience weight discrimination also demonstrate dysregulation of immune, cardiovascular, and metabolic systems and report lower health-related quality of life (QOL) [8,9]. In addition, mortality rates are nearly 60% higher in individuals who experience weight discrimination, even when controlling for other forms of discrimination and physical risk factors [10]. In conjunction with these physical effects, weight discrimination, as well as other forms of weight stigma, can negatively affect mental health. This includes worsened self-esteem, anxiety, depression, suicidality, and eating disorder pathology [11,12,13,14]. In summary, in conjunction with other forms of weight stigma, weight discrimination produces harmful effects on the physiological systems of the body, shortens life expectancy, worsens psychological well-being, and may impact QOL and eating/weight-related psychosocial functioning. It is important to note that while the above discussion has focused on people who are higher weight, weight discrimination can be targeted towards individuals anywhere on the weight spectrum, from lower to higher weight [15]. Thus, it is important to conduct research with people across the weight spectrum.

These negative effects may be influenced by whether or not an individual applies weight discrimination to themselves. The psychological process of internalizing or believing self-devaluing and weight-biased messages and aligning them with one’s identity is referred to as internalized weight stigma [2,16]. Research suggests that 80% of individuals who experience weight discrimination internalize the stigma, while 20% do not [4]. Self-application of negative stereotypes includes believing one is incapable of developing willpower or is inherently ‘lazy’, which in turn can decrease health-promoting behaviors such as exercise [17]. Internalized weight stigma has been found to mediate the relationship between experienced/perceived weight stigma and negative biopsychosocial outcomes [18]. In addition, internalized weight stigma is associated with reduced global sleep quality and daily disturbances related to anxiety and depression [19]. In summary, internalizing weight stigma is widespread, can reduce health-promoting behaviors, and appears to contribute to severe psychological problems, including but not limited to eating disorders [20,21].

Eating disorders (EDs) are, in and of themselves, associated with significant medical complications and elevated morbidity [22], which makes the study of ED pathology and weight stigma of particular importance. Historically, EDs have been stereotypically associated with thin, affluent, young, White women and girls; therefore, researchers and clinicians have not historically viewed individuals with specific demographics such as low income as susceptible to EDs. This focus on affluent, White women has also extended to weight stigma research, leading many researchers to overlook investigating the negative effects of weight stigma in nonaffluent, and racially/ethnically diverse populations. Yet, the limited research available suggests that people with lower levels of education and lower incomes are more likely to report higher levels of internalized weight stigma than the general population [23]. In particular, populations living with food insecurity (FI, i.e., the state of having inadequate access to food of sufficient quantity, quality, and variety) [24,25] report experiencing high levels of internalized weight stigma [20]. Importantly, individuals living with FI have limited availability to nutrient-dense foods and limited accessibility when these foods are available, which may contribute to higher or lower weight and thus greater exposure to all forms of weight stigma.

Though preliminary research suggests that internalized weight stigma coupled with FI is associated with ED pathology [20,26], weight stigma research is scant amongst food-insecure populations. Most recently, Gaston-Panthanki et al. (2023) examined the experiences of bariatric surgery patients and found that those who were living with FI reported higher levels of weight discrimination and internalized weight stigma than those who were food-secure [27]. Qualitative research also suggests that those living with FI internalize broader societal messages that promote the avoidance of higher-weight bodies [28]. For instance, Taylor et al. (2020) found that mother/father dyads reported valuing weight loss stemming from FI as a positive occurrence, thus reflecting the pervasiveness of society’s valuing of thinner bodies [28]. Taken together, these preliminary findings suggest a need to further examine the effects of weight discrimination and stigma in populations living with FI.

The present study sought to extend the above results by examining potential correlates of weight discrimination in a population living with FI. More specifically, this study examined the links between weight discrimination and internalized weight stigma, anxiety, depression, ED pathology, health outcomes, QOL, and eating/weight-related psychosocial impairment in a sample of people living with FI. Investigating the potential effects of weight discrimination in people living with FI is critical for several reasons: Many who work in hunger-relief organizations are concerned about the rates of “obesity” in this underserved community [29,30]. Given the medical correlates of higher weight, such concerns are understandable. Yet, any efforts to address such medical correlates with a weight-normative approach (i.e., one predicated on the idea that thinner bodies are more favorable than heavier ones) run the risk of increasing experiences of weight stigma and potentially weight discrimination (see [31] for additional discussion). As such, it is crucial to begin to document potential correlates of weight stigma and discrimination in this population. An additional reason for conducting this research is that both higher weight and FI disproportionately occur in racial and ethnic minority communities [32,33]. Given their intersectional disadvantages, the probability of experiencing outright weight discrimination is higher in these communities [5].

It is important to note that we are proposing and concerned about longitudinal relationships; yet, the present study is correlational. Thus, we could not fully address the set of relationships described above, which would necessitate a longitudinal design. Longitudinal studies, however, are more expensive to run and procuring such funding requires evidence of the proposed relationships. Thus, this study was designed to serve as foundational research needed to support future longitudinal research should our hypotheses be supported.

Based on past research with food-secure people, we hypothesized that experiences of weight discrimination would be associated with higher levels of internalized weight stigma, anxiety, depression, ED pathology, negative health outcomes, and eating/weight-related psychosocial impairment. In addition, we hypothesized that experiences of weight discrimination would be associated with lower levels of QOL. Finally, we hypothesized that internalized weight stigma and poorer mental health (i.e., increased depression, anxiety, and ED pathology) would cross-sectionally mediate the association of weight discrimination with QOL, health outcomes, and eating/weight-related psychosocial impairment.

## 2. Materials and Methods

### 2.1. Participants

The sample consisted of 1085 adult clients (age *M* = 48.81, *SD* = 15.95) of the San Antonio Food Bank (SAFB). Participants identified as predominantly women (69.1%), followed by men (29.7%); 7 participants identified as nonbinary, 3 selected other, and 3 participants did not answer. Similar to the San Antonio population, nearly 78% of the sample identified as Hispanic/Latino [34]. Approximately half (48.9%) of our sample had an annual income of less than USD 10,000. Most participants had achieved a high school diploma or less (93%). The mean household size was 3.5 (*SD* = 2.18). For participants who had children, the mean number of children in the home was 1.07 (*SD* = 1.36; see Table 1 for additional demographics).

### 2.2. Procedure

This study was approved by the Institutional Review Board (IRB) at Trinity University and was conducted in collaboration with the SAFB. The SAFB serves 29 counties in Texas and approximately 90,000 individuals each week. According to a USDA report, 13.3% of Texans experience FI [25]. Almost 20% of people living in San Antonio live below the official poverty measure as established by the U.S. Census Bureau [35].

Research assistants (RAs) collected data at the SAFB headquarters in the waiting area for both Client Services and Workforce Solutions Alamo. At Client Services, individuals can receive assistance with a variety of tasks including completing applications for federal benefits; Workforce Solutions Alamo helps individuals find employment information and provides job training options. Although the primary function of the SAFB is to distribute food, this location also provides assistance with federal benefits and employment. It should be noted that the clients who were recruited for this study were not there to receive food. Clients often wait 30 to 50 min to be seen by the staff of Client Services or Workforce Solutions Alamo. During this waiting period, RAs approached SAFB clients to ask if they were interested in possibly participating in the study using a standardized recruitment script. If the client agreed, the RAs provided more detailed information about the study. To accommodate language preferences of the participants, all scripts and materials were available in both English and Spanish. Spanish-speaking RAs were present throughout the data collection period.

Subsequently, participants were presented with a consent form, which they could either read independently or have the RAs provide a summary of each component. Upon signing the consent form, participants were given either a paper or tablet version of the survey based on individual preference. Both the paper and tablet version were coded numerically to organize the data without collecting identifying information about participants. To further maintain confidentiality, the consent form was kept separate from the survey. Participants were asked not to include their name or any other identifying information on the questionnaire. On average, the survey took 25 min to complete.

RAs read survey questions aloud and helped record the responses for participants who had difficulty reading. If the participant was called into their appointment with Client Services or Workforce Solutions Alamo, the RAs kept their survey and asked if they wanted to continue after the appointment. Participants signed for and received a gift card (USD 8) to a local grocery store chain upon survey completion. RAs debriefed each participant after completion and provided them with a copy of the consent form along with a list of low-cost/free mental health resources. Data collection took place in April of 2022 through July of 2022.

### 2.3. Translation

Given the large percentage of Spanish-speakers in San Antonio, we provided a Spanish version of our materials for those who wanted them. As the majority of San Antonio’s Hispanic/Latino community is of Mexican descent, we sought measures that had been translated into the Spanish dialect spoken in Mexico, such as the GAD-7 and PHQ-8 [36]. For questionnaires that did not have a validated translation, items were translated by two bilingual RAs, and a back-translation was performed by two other bilingual RAs (e.g., WSSQ). To ensure linguistic accuracy, a dialect check was then conducted by a bilingual individual who has resided in San Antonio for the majority of their life. Based on their recommendations, minor adjustments were made to align the translated materials with the Mexican-based dialect of the Spanish speaking community in San Antonio.

### 2.4. Measures

#### 2.4.1. Demographics

Participants were asked to provide a variety of demographic information, including their age, gender, sexual identity/orientation, language spoken at home, marital status, household size, number of children in the home, and any government benefits they were receiving (Table 1).

#### 2.4.2. Food Insecurity Status

Radimer/Cornell Food Insecurity Measure (RCFIM). Food insecurity (FI) was measured using the RCFIM [37,38]. This is a 13-item measure with responses on a 3-point Likert scale (0 = not true, 1 = sometimes true, 2 = always true). Sample items include “*I worry where my next day’s food is going to come from*” and “*I can’t afford to eat properly*”. Individuals are classified as one of the following: food-secure, household FI, individual FI, or child hunger FI. Given that adults typically attempt to shield children from hunger, child hunger FI implies that adults present in the household are even hungrier [39]. Thus, the last category is the most severe level of FI. We modified the food secure category to be “not food insecure”. Despite not meeting the criteria for FI, participants were visiting the food bank for assistance, which indicates that they may be living on the margins of food security and insecurity.

We followed the standard scoring instructions to determine the level of FI of participants. Selection of “Not True” for all items indicated a participant was not food insecure. The RCFIM contained three main types of questions: lowest severity (e.g., worrying about food), higher severity (e.g., reported going hungry), and child hunger (e.g., reported children in the home going hungry). Those who select “Sometimes True” or “Always True” for the lowest severity items but “Not True” for the two higher categories were considered household FI. Participants in this FI group often feel anxious about having access to food and eat the same food repeatedly due to lack of access or not being able to afford a diverse diet. Participants who selected “Sometimes True” or “Always True” for the higher severity items but “Not True” for the child hunger items were considered individual FI. These participants were reporting not eating enough and going hungry due to a lack of access to food. If participants endorsed any of the child hunger items, then they were considered child hunger FI.

We selected the RCFIM instead of the U.S. Department of Agriculture Food Security Scale (USDA). The RCFIM is shorter in length and is relatively easier to read than the USDA measure, which is important given the expected low education level of our sample. The RCFIM has strong internal consistency as well as construct and criterion validity [38]. We found favorable internal consistency in our sample (Cronbach’s *α* = 0.834).

#### 2.4.3. Weight Discrimination and Stigma

Experiences of Weight-Based Discrimination (EWBD). Participants were asked to rate the extent to which they had experienced weight-based discrimination using the EWBD measure. The EWBD is based on the Schmitt et al.’s (2003) gender-based discrimination scale but was modified by Farrow and Tarrant (2009) to relate to perceptions of weight discrimination (“*I regularly encounter weight related discrimination*” *and* “*Prejudice against overweight people has affected me personally*”) [40,41]. The EWBD consists of 6 items that are rated on a 7-point Likert scale (1 = totally disagree to 7 = totally agree). Scores on the EWBD consist of a sum of all the items. The 6 items had excellent internal consistency within our sample (Cronbach’s α = 0.963).

Weight Self-Stigma Questionnaire (WSSQ). Internalized weight stigma was measured using the WSSQ [42]. The WSSQ is a 10-item scale that uses a 5-point Likert scale (1 = completely disagree to 5 = completely agree). Sum scores are calculated for the total scale and two subscales. Items 1–6 compose the self-devaluation subscale, and items 7–10 determine the fear of enacted stigma subscale. Consistent with two of our previous studies [26,43], we modified questions to make it more comprehensible for participants with lower reading levels. Two items from the original measure (twelve items) were removed due to the complexity of the questions, and we changed the wording of “weight problems” and “overweight” to “fat” (e.g., “*People discriminate against me because I’m fat*”). The measure has excellent internal validity in our sample (Cronbach’s *α* = 0.944) and acceptable face validity [44]. It is critical that marginalized populations are understood and valued, which justifies the alteration of the wording in questionnaires [45]. Therefore, these language changes were necessary to reach this understudied population.

#### 2.4.4. Eating Disorder Pathology

Eating Disorder-15 (ED-15). Eating disorder pathology was measured using the ED-15. This 15-item questionnaire measures the frequency of eating disorder symptoms over the past week on a 7-point Likert scale (0 = not at all to 6 = all of the time). A sample item includes “*avoided activities or people because of the way I look*”. The first ten items comprise attitudinal items, and the final five items are only answered if participants have engaged in maladaptive behaviors such as purging to control weight or shape. These items require open-ended responses with one of the items being: “*on how many days in the past week have you used laxatives to control your weight or shape?*” We used the standard scoring of the ED-15, which involves calculating a mean of the scores on the attitudinal items. The ED-15 has good psychometric properties and clinical validity [46]. In the current sample, the ED-15 had excellent internal validity (Cronbach’s *α* = 0.924).

#### 2.4.5. Eating- and Weight-Related Psychosocial Impairment

Clinical Impairment Assessment Questionnaire (CIAQ). The CIAQ was used to measure eating- and weight-related psychosocial impairment. This 16-item questionnaire asks about the impact of exercising, eating habits, and feelings about weight/shape on various aspects of life such as mood, cognitive functioning, social engagement, and work performance using a 4-point Likert scale (0 = not at all to 3 = a lot). The CIAQ score is the sum of all 16 items on the scale. A sample item is “*over the past 28 days, to what extent have your eating habits, exercising, or feelings about your eating, shape or weight affected your ability to make everyday decisions?*” The CIAQ has very good internal consistency and construct validity [47]. It also had excellent internal validity in our sample (current Cronbach’s *α* = 0.976).

#### 2.4.6. Anxiety

Generalized Anxiety Disorder questionnaire (GAD-7). Anxiety was assessed employing the GAD-7. We chose this measure of anxiety for its brevity and efficiency. This 7-item questionnaire measures the frequency of anxiety symptoms on a 4-point Likert scale (0 = not at all to 3 = nearly every day). Participants are asked to reflect on the last two weeks and indicate the extent to which they have felt nervous, anxious, on edge, or had trouble relaxing. Research suggests that the GAD-7 is a valid and reliable measure of anxiety (Cronbach’s *α* = 0.92) [48]. Spitzer and colleagues (2006) also reported good construct and criterion validity [48]. Within our sample, the GAD-7 had excellent internal validity (Cronbach’s *α* = 0.90).

#### 2.4.7. Depression

Personal Health Questionnaire Depression Scale (PHQ-8). The PHQ-8 was used to measure depressive symptoms. Participants are asked to reflect on the preceding two weeks and rate how often they were bothered by issues such as experiencing little interest or pleasure in doing things [49]. Items were rated on a 4-point Likert scale (0 = not at all to 4 = nearly every day). The PHQ-8 is a well-validated and reliable measure of depressive symptoms (Cronbach’s *α* = 0.88) [50,51]. Research supports satisfactory criterion and construct validity [49].

The PHQ-8 was selected over the 9-item version (PHQ-9) because the PHQ-8 excludes the final item pertaining to suicidal ideation (“*Thoughts that you would be better off dead, or of hurting yourself*”). The PHQ-8 and the PHQ-9 are highly correlated (r = 0.997) and have similar sensitivity [52]. The PHQ-8 had excellent internal validity in our sample (Cronbach’s *α* = 0.924).

#### 2.4.8. Health Outcomes

For health outcomes, we asked participants if they had ever been diagnosed with diabetes, prediabetes, hypertension, cancer, high cholesterol, and/or a heart attack. These were yes or no questions (0 = no, 1 = yes). Each diagnosis received a score of one, but diagnoses of prediabetes and diabetes were combined as a single diagnosis. Therefore, when a participant reported being diagnosed with both diabetes and prediabetes, they received a score of one to avoid inflating their score. The scores were summed to obtain a composite score for health outcomes that ranged from 0 to 5.

#### 2.4.9. Quality of Life (QOL)

EUROHIS-QOL-8. QOL was measured using the EUROHIS-QOL-8. This is an 8-item measure consisting of items extracted from the World Health Organization Quality of Life Scale (WHOQOL-BREF). These eight items measure overall quality of life, general health, energy, daily living activity, self-esteem, social relationships, finances, and living conditions for the past two weeks [53]. A sample item from the EUROHIS-QOL-8 includes “*How satisfied are you with your ability to perform your daily living activities?*” Responses are recorded on a 5-point Likert scale with varying response wording depending on the item (e.g., 1 = very poor to 5 = very good; 1 = not at all to 5 = completely). This QOL instrument is drastically shorter than other QOL measures; we purposely selected this measure to ease the response burden for our participants. The EUROHIS-QOL-8 has good cross-cultural internal consistency (Cronbach’s *α* ranging from 0.72 to 0.81 across 6 countries) [54]; the EUROHIS-QOL-8 had excellent internal validity in the current sample (Cronbach’s *α* = 0.872). Furthermore, research suggests acceptable discriminant and convergent validity [54,55].

### 2.5. Data Analysis

Our first aim was to examine the correlations between weight discrimination, health, psychological outcomes, QOL, and eating/weight-related psychosocial impairment. Thus, we conducted bivariate correlation analyses to explore the relationship between weight discrimination, internalized weight stigma, depression, anxiety, ED pathology, health outcomes, QOL, and eating/weight-related psychosocial impairment. Our second aim was to analyze the role of internalized weight stigma and negative psychological outcomes (i.e., depression, anxiety, ED pathology) as potential cross-sectional mediators in the relationship between weight discrimination and health outcomes, QOL, and eating/weight-related psychosocial impairment. We conducted several serial mediation analyses using PROCESS for SPSS [56] based on significant bivariate correlation analyses. Each serial mediation included internalized weight stigma as the first mediator, a mental health indicator (i.e., anxiety, depression, or ED pathology) as the second mediator, and QOL or eating/weight-related psychosocial impairment as the outcome variable.

To address missing data in the measures of anxiety, QOL, weight stigma, depression, and internalized weight stigma, we imputed an average score value if the participant was only missing one item of the scale. For the measure of ED pathology, the participant could miss a maximum of one item for each subscale, which was imputed with the overall average score. Finally, for the measure of eating/weight-related psychosocial impairment, participants could miss a maximum of four items, which was imputed with the overall average score. For participants missing additional items on a scale, their data were considered invalid and were not included in the analyses.

## 3. Results

### 3.1. Experiences and Correlates of Weight Discrimination

Overall, 23.2% of our sample endorsed experiencing weight discrimination. Participants most commonly reported personally being a victim of weight discrimination over other statements (Table 2). Interestingly, a one-way ANOVA comparing EWBD scores between FI groups revealed a significant difference across FI groups [*F*(3, 1056) = 13.16, *p* < 0.001]. Post hoc analyses using Tukey’s HSD test revealed that the mean level of weight discrimination was higher in the individual FI group (*M* = 13.00, *SD* = 9.32) and the child hunger FI group (*M* = 14.63, *SD* = 10.44) than in the two lower levels of FI, household FI (*M* = 9.68, *SD* = 5.93) and not food insecure (*M* = 8.87, *SD* = 6.53). These results suggest that those who face the highest levels of FI are experiencing higher levels of weight discrimination.

Regarding our first aim, weight discrimination was significantly correlated with anxiety, depression, internalized weight stigma, ED pathology, QOL, and eating/weight-related psychosocial impairment (see Table 3). However, weight discrimination was not significantly correlated with health outcomes; thus, we limited our mediational analyses to include two main outcomes: QOL and eating/weight-related psychosocial impairment. Experiences of weight discrimination were associated with higher levels of internalized weight stigma, anxiety, depression, ED pathology, and eating/weight-related psychosocial impairment, as well as lower QOL.

### 3.2. QOL as Main Outcome

**Figure 1:**
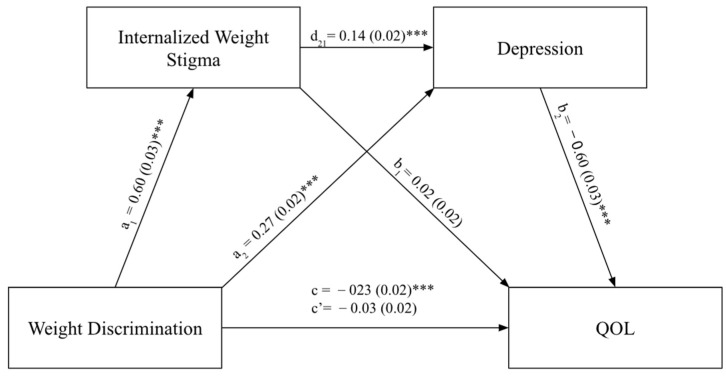
Internalized weight stigma and depression mediating the relationship between weight discrimination and QOL. Note: *** *p* < 0.001; QOL refers to quality of life. a_1_ refers to the regression of weight discrimination on internalized weight stigma. a_2_ refers to regression of weight discrimination on depression. d_21_ refers to regression of internalized weight stigma on depression controlling for weight discrimination. b_1_ refers to regression of internalized weight stigma on quality of life controlling for weight discrimination. b_2_ refers to regression of depression on quality of life controlling for weight discrimination. c refers to the total effect and c’ refers to the direct effect.

Regarding the results of the serial mediation model using depressive symptoms as the second mediator, there was a significant indirect effect of weight discrimination on QOL through internalized weight stigma and depression (b = −0.05, SE = 0.009, 95% CI (−0.07, −0.03)). A direct effect of weight discrimination on QOL, without the presence of the mediators, was nonsignificant (b = −0.03, *t*(1016) = −1.67, *p* = 0.10). Thus, the relationship between weight discrimination and QOL was fully mediated by internalized weight stigma and depression (see Figure 1 for additional details).

In addition to depressive symptoms, serial mediation analyses were conducted to identify the role of anxiety and ED pathology in the association between weight discrimination and QOL. The mediation analyses conducted for ED pathology and anxiety resemble the model shown in Figure 1, with ED pathology and anxiety replacing depression as second mediators. For the model including anxiety, we found an indirect effect of weight discrimination on QOL through internalized weight stigma and anxiety (b = −0.02, SE = 0.007, 95% CI (−0.04, −0.01)). A direct effect of weight discrimination on QOL, without the presence of the mediators, was also found (b = −0.10, *t*(1014) = −4.99, *p* < 0.001). Therefore, the relationship between weight discrimination and QOL was partially mediated by internalized weight stigma and anxiety. Finally, we found an indirect effect of weight discrimination on QOL through internalized weight stigma and ED pathology (b = −0.05, SE = 0.008, 95% CI (−0.07, −0.04)). Yet again, the direct effect of weight discrimination on QOL, without the presence of the mediators, was significant (b = −0.06, *t*(1022) = −2.43, *p* = 0.02]. Thus, the relationship between weight discrimination and QOL was partially mediated by internalized weight stigma and ED pathology.

### 3.3. Eating/Weight-Related Psychosocial Impairment as the Main Outcome

Similar to the three mediation analyses conducted for QOL as the main outcome, the same data analyses process was implemented for eating/weight-related psychosocial impairment as the main outcome. Depression, anxiety, and ED pathology were investigated as potential mediators in the relationship between weight discrimination and eating/weight-related psychosocial impairment. The indirect effect of weight discrimination on eating/weight-related psychosocial impairment through internalized weight stigma and depression was significant (b = 0.07, SE = 0.014, 95% CI (0.05, 0.10)). The direct effect of weight discrimination on eating/weight-related psychosocial impairment, without the presence of the mediators, also was significant (b = 0.53, *t*(1007) = 15.47, *p* < 0.001). Thus, the serial mediation partially explains the relationship between weight discrimination and eating/weight-related psychosocial impairment.

Regarding anxiety, the mediation model demonstrated an indirect effect of weight discrimination on eating/weight-related psychosocial impairment through internalized weight stigma and anxiety symptoms (b = 0.03, SE = 0.011, 95% CI (0.01, 0.06)). The direct effect of weight discrimination on eating/weight-related psychosocial impairment, without any mediators, was significant (b = 0.62, *t*(1006) = 17.84, *p* < 0.001]. Therefore, the relationship between weight discrimination and eating/weight-related psychosocial impairment was partially mediated by internalized weight stigma and anxiety symptoms.

**Figure 2:**
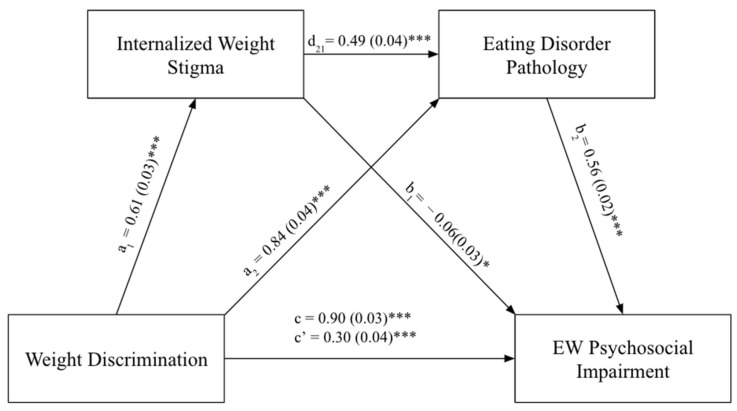
Internalized weight stigma and eating disorder pathology mediating the relationship between weight discrimination and EW psychosocial impairment. Note. * *p* < 0.05, *** *p* < 0.001; EW psychosocial impairment refers to eating/weight-related psychosocial impairment. a_1_ refers to the regression of weight discrimination on internalized weight stigma. a_2_ refers to regression of weight discrimination on eating disorder pathology. d_21_ refers to regression of internalized weight stigma on eating disorder pathology controlling for weight discrimination. b_1_ refers to regression of internalized weight stigma on EW psychosocial impairment controlling for weight discrimination. b_2_ refers to regression of eating disorder pathology on EW psychosocial impairment controlling for weight discrimination. c refers to the total effect. c’ refers to the direct effect.

Finally, there was a significant indirect effect of internalized weight stigma and ED pathology mediating the effect of weight discrimination on eating/weight-related psychosocial impairment (b = 0.17, SE = 0.022, 95% CI (0.13, 0.21)). The direct effect of weight discrimination on eating/weight-related psychosocial impairment, without any mediators, was significant (b = 0.30, *t*(1013) = 8.38, *p* < 0.001). Therefore, the relationship between weight discrimination and eating/weight-related psychosocial impairment was partially mediated by internalized weight stigma and ED pathology (see Figure 2).

## 4. Discussion

The primary aim of this study was to identify correlates of weight discrimination in a population living with FI. We hypothesized that individuals living with FI would report experiencing weight discrimination. Indeed, 23.2% of our sample reported experiencing at least one form of weight discrimination. The rates of weight discrimination in our sample are much higher than those of a national U.S. sample, where 10.3% of women and 4.9% of men reported daily or lifetime experiences of weight discrimination [4]. This indicates that individuals experiencing FI may experience more frequent weight discrimination than the general population.

The relationship between FI and weight discrimination may be due to the high prevalence of higher weight bodies in food-insecure populations; in the U.S., those living with FI tend to have greater access to energy-dense, processed foods rather than nutrient-dense foods [57,58]. Furthermore, to understand the longitudinal relationship between FI and weight discrimination, Martinez-Jaikel (2017) proposed a theoretical framework of how weight discrimination impacts a person through economic domains that lead to FI [59]. Weight discrimination can occur in employment and education settings that may lead to negative outcomes such as less job opportunities, worse treatment in employment, or lower educational attainment [60,61,62]. According to Martinez-Jaikel (2017), these limited employment and educational opportunities, caused by weight discrimination, may then contribute to FI [59]. Our study is one of few to document the high prevalence of weight discrimination among those who are food-insecure.

We sought to explore how weight discrimination is linked to QOL and eating/weight-related psychosocial impairment. Our results suggest that experiences of weight discrimination were associated with higher levels of internalized weight stigma, anxiety, depression, ED pathology, eating/weight-related psychosocial impairment, and lower QOL. This aligns with previous research suggesting that experiencing weight discrimination is associated with poorer mental health in post-bariatric-surgery patients [27]. A meta-analysis also suggests that all forms of weight stigma are negatively associated with mental health [11].

Additionally, in our study, the relationship between weight discrimination and QOL was fully mediated by internalized weight stigma and depression. Importantly, as previously mentioned, while our study includes urban participants living with FI who have greater access to energy-dense and processed foods and may have higher weights, weight discrimination can be targeted towards individuals across the weight spectrum [5]. As individuals experience weight discrimination, they may internalize those negative beliefs and apply them to themselves which may lead to depressive symptoms and contribute to a lower QOL. Longitudinal research will need to confirm if this proposed relationship holds up in the proposed direction. All other mediation models resulted in internalized weight stigma and mental health indicators partially mediating the relationship between weight discrimination and QOL or eating/weight-related psychosocial impairment. These results begin to explain the association between weight discrimination and QOL as well as eating/weight-related psychosocial impairment.

Of note, weight discrimination was not significantly correlated with health outcomes. However, our measurement of health was extremely limited, which could have contributed to this null finding. As previously mentioned, weight discrimination has been linked to greater mortality risk as well as higher levels of inflammatory markers, specifically C-reactive protein, which is a predictor of diabetes and cardiovascular disease [10,63]. Weight discrimination may affect an individual’s health, but our measure was not able to capture it.

The prevalence of weight discrimination is a concerning issue in society, particularly among those in low-income, food-insecure populations. This population faces the dual burden of not having access to food along with the negative societal attitudes and judgements associated with their weight. Individuals who are food-insecure often face weight-stigmatizing messages and are subject to weight discrimination, which are likely to further harm their mental health and QOL while worsening eating/weight-related psychosocial impairment. Furthermore, weight stigma in childhood was linked to the risk of higher weight in adulthood [64], and there are no reasons to assume this finding would not extend to children who are food-insecure.

### Implications and Recommendations

Identifying the correlates of weight discrimination in food-insecure individuals has important clinical implications, as it might allow us to reconsider the language that is used in public health messages and medical settings. In fact, a recent study conducted by Mensinger and colleagues (2023) examined the effects of weight-inclusive health promotion programs in addressing maladaptive eating behaviors [65]. They suggested that weight-inclusive health promotion programs that focus on reducing internalized weight stigma can improve maladaptive eating patterns. Another treatment that targeted women of higher weight status indicated that post-treatment improvements in QOL and health-related outcomes were mediated by a decrease in internalized weight stigma [66]. Both of these treatments demonstrate a need to address internalized weight stigma as a means of improving maladaptive eating behaviors and enhancing an individual’s QOL. However, it is also necessary to consider the experiences of weight discrimination that are pervasive in society and are contributing to the internalization of weight stigma. Pearl (2018) suggests policy changes to combat weight discrimination as well as developing trainings to reduce weight bias in educational and medical settings as potential avenues for addressing weight discrimination on a population level [67].

Regarding limitations, our measurement of health outcomes was limited to a composite score of current health diagnoses. Considering that this is a marginalized population with limited access to healthcare, they may be experiencing undiagnosed health concerns. Thus, having a more robust assessment of health outcomes could help improve the validity of our findings. Furthermore, we did not specifically collect data for BMI in our study. BMI data could have been useful in understanding if the link between weight discrimination and FI could be due to higher weight as well as how food-insecure individuals of different body weights experience internalized weight stigma. However, given the limited access to physicians and at-home scales, self-reports of height and weight would likely not be accurate. Weighing participants in a public area would have been insensitive and could potentially contribute to weight stigma. While the BMI is used to categorize individuals by weight, it has little validity as a health indicator; it was also designed using a White, Western population, so is not generalizable to our sample [68,69]. Additionally, our measure of ED pathology is intended to measure ED symptoms over the last week. Given that the cyclical nature of FI (i.e., greater access and availability to food at the beginning of the month but food scarcity towards the end of the month) can be associated with greater ED behaviors at certain times of the month [70], our measure may not be able to capture the full extent of ED symptoms that an individual experiences over a longer period of time. It is also important to mention that we do not expect our findings to reflect an inflation of ED behaviors because participants were at the food bank to receive assistance in applying for or renewing federal benefits rather than to receive food assistance. Lastly, this study is cross-sectional and as such, no direct causation between weight discrimination, internalized weight stigma, QOL, eating/weight-related psychosocial impairment, and FI can be inferred from our analyses.

Future research should aim to expand the scope of research beyond the Hispanic/Latino community to include other food-insecure populations to enhance generalizability of our findings. In addition, conducting longitudinal studies would capture the long-term impact of weight discrimination on QOL, eating/weight-related psychosocial impairment, and mental and physical health. To improve the effectiveness of health intervention, future studies should develop and pilot weight-neutral interventions. Lastly, it is important to identify factors that may be protective against internalizing weight stigma after experiencing weight discrimination in order to support communities that are more affected by these experiences.

## 5. Conclusions

In our study, almost one out of four people reported having experienced weight discrimination. Evidently, food-insecure populations are experiencing weight discrimination at elevated rates that are associated with lower QOL and psychosocial functioning, particularly through internalized weight stigma and mental health. It is critical to recognize that internalized weight stigma and weight discrimination are risks for psychological and physiological well-being. Our large sample size demonstrates the impact of weight discrimination in FI communities and can help advocate for the importance of taking actionable steps to reduce weight discrimination. By advocating for less-stigmatizing terminology and messages, such as using “*higher body weight*” instead of “*obese*” (this term itself is viewed by many as stigmatizing due to the negative medical stigma surrounding it), we can reduce the amount of internalized weight stigma and promote nutrition and health to all communities. Adopting a holistic approach to health is critical in addressing the unique needs and challenges faced by food insecure populations. By taking into account the intersection of FI, weight discrimination, and overall health and well-being, we can reduce barriers to an improved quality of life.

## Figures and Tables

**Table 1:** Demographics of samples with food-insecurity levels.

	Total Sample (*N* = 1085)		Not Food Insecure (*n =* 69)		Household Food Insecure (*n =* 111)		Individual Food Insecure (*n =* 482)		Child Hunger Food Insecure (*n =* 423)	
	*n*	%	*n*	%	*n*	%	*n*	%	*n*	%
*Sex*										
Female	750	69.1	41	59.4	88	79.3	309	64.1	312	73.8
Male	322	29.7	28	40.6	23	20.7	166	34.4	105	24.8
*Education*										
Grade school or less	139	12.8	5	7.2	14	12.6	48	10.0	29	6.9
Some high school	225	20.7	17	24.6	26	23.4	84	17.4	98	23.3
High school diploma	415	38.2	21	30.4	40	36.0	192	39.8	162	38.3
Some college	230	21.2	15	21.7	19	17.1	103	21.4	98	22.0
College graduate/ postgraduate	75	6.9	9	13.0	9	8.1	36	7.4	21	4.9
*Race/Ethnicity*										
Hispanic/Latino	844	77.8	59	85.5	93	83.8	362	75.1	330	78.0
Black/African American	52	4.8	2	2.9	4	3.6	23	4.8	23	5.4
White	105	9.7	5	7.2	7	6.3	63	13.1	30	7.1
Other	78	7.2	3	4.3	6	5.4	32	6.6	37	8.8
*Annual Household Income (USD)*										
≤10,000	531	48.9	22	31.9	47	42.3	241	50.0	221	52.2
10,000–20,000	307	28.2	18	26.1	37	33.3	148	30.7	104	24.6
20,000–30,000	95	8.8	2	2.9	8	7.2	39	8.1	46	10.9
30,000–40,000	49	4.5	5	7.2	7	6.3	20	4.1	17	4.0
>40,000	51	4.8	16	23.1	8	7.2	12	2.4	15	3.5
*Sexual Orientation*										
Heterosexual	899	82.9	57	87.7	92	82.9	404	83.8	346	81.8
Bisexual	35	3.2	1	1.4	4	3.6	13	2.7	17	4.0
Lesbian/Gay	32	2.9	4	5.8	6	5.4	18	3.7	4	0.9
Other	38	3.5	3	4.3	1	1.0	5	1.0	5	1.2
*Language Spoken at Home*										
English	613	56.5	41	59.4	49	44.1	298	61.8	225	53.2
Spanish	304	28.0	18	26.1	38	34.2	110	22.8	138	32.6
Both Equally	154	14.2	10	14.5	22	19.8	67	13.9	55	13.0
*Marital Status*										
Single	435	40.1	28	40.6	38	34.2	208	43.2	161	38.1
Married/Living with Partner	334	30.8	19	27.5	50	45.0	118	24.5	147	34.8
Separated	111	10.2	6	8.7	7	6.3	46	9.5	52	12.3
Divorced	114	10.5	9	13.0	8	7.2	59	12.2	38	9.0
Widowed	89	8.2	7	10.1	7	6.3	51	10.6	24	5.7
*Employment*										
Unemployed	443	40.8	19	27.5	32	28.8	196	40.7	196	46.3
Disabled	248	22.9	8	11.6	15	13.5	142	29.5	83	19.6
Full-time	154	14.2	14	20.3	22	19.8	50	10.4	68	16.1
Part-time	157	14.5	9	13.0	17	15.3	69	14.3	62	14.7
Retired/ homemaker	179	16.5	20	29.0	30	27.0	86	17.8	43	10.2
Student (FT/PT)	31	2.9	3	4.3	2	0.2	15	3.2	11	2.6
*Government Benefits*										
Social Security	266	24.5	21	30.4	35	31.5	155	32.2	55	13.0
SSI	146	13.5	5	7.2	11	9.9	79	16.4	51	12.1
Medicare	209	19.3	11	15.9	28	25.2	101	21.0	69	16.3
Medicaid	192	17.7	8	11.6	25	22.5	97	20.1	62	14.7
CHIP	174	16.0	8	11.6	26	23.4	29	6.0	111	26.2
SNAP	383	35.3	17	24.6	54	48.6	161	33.4	151	35.7
WIC	65	6.0	1	1.4	21	18.9	14	2.9	29	6.9

Note. Individuals could report multiple government benefits. Government benefits reported by less than 5% of the sample included TANF, Section 8 Housing Program, Unemployment benefits, Veteran’s benefits, CEAP, Women’s Health Program, Long-Term Care.

**Table 2:** Response rate for each item of the EWBD.

EWBD Items	Totally Disagree (%)	Disagree (%)	Somewhat Disagree (%)	Neither Agree, Nor Disagree (%)	Somewhat Agree (%)	Agree (%)	Agree Very Much (%)
I have personally been a victim of weight-related discrimination	51.9	17.1	6.5	7.7	5.8	4.4	4.4
I consider myself a person who has been deprived of opportunities because of my weight	53	18.9	6.3	6.6	5.3	3.5	4.2
I feel like I am personally a victim of society because of my weight	54.5	18.9	4.8	8.1	5	2.9	3.8
I have personally been a victim of weight-related harassment	54.5	18	5.6	6.8	4.9	3.7	4.4
I regularly encounter weight-related discrimination	54.5	19.3	5.3	7.3	5	3.3	3
Prejudice against overweight people has affected me personally	53.8	18.6	5.2	6.9	4.4	4.1	4.6

Note. EWBD refers to experiences of weight-based discrimination.

**Table 3:** Correlations between all variables.

	M	SD	1	2	3	4	5	6	7	8
1. EWBD	13.0	9.5	—							
2. IWS	22.4	10.7	0.531 *	—						
3. Anxiety	8.7	6.5	0.361 *	0.285 *	—					
4. Depression	8.4	6.7	0.490 *	0.414 *	0.725 *	—				
5. Eating Disorders	13.6	15.7	0.686 *	0.613 *	0.497 *	0.630 *	—			
6. Health Outcomes	1.3	1.4	0.016	0.013	0.028	0.049	0.011	—		
7. Quality of Life	24.9	6.3	−0.358 *	−0.269 *	−0.576 *	−0.660 *	−0.454 *	−0.113 *	—	
8. Psychosocial Impairment	10.5	13.0	0.655 *	0.483 *	0.573 *	0.686 *	0.794 *	−0.015	−0.562 *	—

Note. EWBD refers to experiences of weight-based discrimination, and IWS refers to internalized weight stigma. * indicates *p* < 0.001

## Data Availability

The data presented in this study are available on request from the senior author (Carolyn Becker; cbecker@trinity.edu).
